# Short-term effects of ophthalmic topical 0.01% atropine on the ocular surface, pupil size, and subsequent subjective quality of vision in young myopic Chinese adults

**DOI:** 10.3389/fmed.2024.1436551

**Published:** 2024-09-05

**Authors:** Mingze Li, Yimeng Chen, Jiayan Chen, Guanghao Qin, Liangzhe Li, Wei He, Sile Yu, Xingru He, Emmanuel Eric Pazo, Ling Xu

**Affiliations:** ^1^He Eye Specialist Hospital, Shenyang, China; ^2^The Second Affiliated Hospital, Dalian Medical University, Dalian, China; ^3^The Third Affiliated Hospital, Jinzhou Medical University, Jinzhou, China; ^4^School of public health, He University, Shenyang, China

**Keywords:** atropine, pupil size, quality of vision, dry eye, OSDI questionnaire

## Abstract

**Background:**

Daily use of low concentrations of atropine is recommended for children undergoing myopia control therapy. While the benefits of controlling myopia progression have been confirmed, the potential unwanted side effects on the ocular surface, pupil size, and quality of vision following the administration of 0.01% atropine have not been investigated.

**Objective:**

This single-arm, self-control study aimed to investigate the short-term effects of 0.01% atropine topical eye drop (He Eye Hospital Co., Ltd., Shenyang, China) on pupil size and subjective quality of vision in participants with myopia. Each 3 mL vial of eye drops contains atropine (0.01%), sodium chloride (0.9%), and benzalkonium chloride (0.005%) in an aqueous solution.

**Methods:**

Thirty-three adults (66 eyes) were recruited for the study. The mean age of the participants recruited for this study was 24.91 ± 3.36 years. This study is registered with Clinical Trials.gov (NCT06071260). Assessments were performed at baseline and 10 h, 14 h, and 18 h following the administration of 0.01% topical atropine drop (TAD). Mesopic pupil diameter (MPD), photopic pupil diameter (PPD), higher order aberration (HOA), non-invasive tear breakup time (NITBUT), tear meniscus height (TMH), tear film lipid layer (TFLL), and Redness score (RS). Subjective assessments included the quality of vision (QoV) and the ocular surface disease index (OSDI) questionnaires.

**Results:**

Following the use of 0.01% atropine, PPD significantly increased at all the time points (*p* < 0.001); MPD increased significantly at 10 h and 14 h (*p* < 0.001 and *p* < 0.05, respectively). A decrease in TMH and an increase in the OSDI questionnaire scores were observed up to 10 and 14 h, respectively, after using atropine (*p* < 0.001). Glare (*p* = 0.004 at 10 h and *p* = 0.003 at 14 h), blurred vision (*p* < 0.0001 at 10 h and *p* = 0.035 at 14 h), and focusing difficulties (*p* < 0.0001 at 10 h and *p <* 0.0001 at 14 h) were significantly higher at both 10 h and 14 h after using atropine. No significant changes were observed in the HOA, NITBUT, and RS scores (all *p* > 0.05) at all time points.

**Conclusion:**

Decreased TMH, dry eye symptoms, and visual symptoms will likely persist overnight but often diminish within 18 h after using 0.01% atropine eye drops.

## Highlights

- Both subjective and objective signs and symptoms of dry eye were evaluated, and measurements of pupil size and visual quality were taken before and after the use of 0.01% atropine.
- Objective instrumental measurements and subjective questionnaires were used for data collection.
- The sample size was not large enough, and bias may have occurred.
- Lack of corneal fluorescence staining to assess corneal status.
- The present study specifically examined myopic individuals in their youth, rather than children.
- The study assessed short-term immediate changes; a longer-term follow-up study is warranted.

## 1 Introduction

Myopia has become a global health issue due to its growing prevalence, earlier onset, and progression (1, 2). Myopia progression is significantly linked to eye Axial length (AL) (3–5). Several treatments, including low-concentration topical atropine eye drops, have been reported to significantly slow down axial elongation (6, 7). While the safety and efficacy of topical atropine have been documented to be optimal, adverse events have not been stringently reported by all articles (8–10). As stated by North et al. in 1987, there are a myriad of adverse effects (AEs) related to topical administration of atropine (11).

Furthermore, the effect of atropine on the progression of myopia is proportional to its concentration, with greater effects and more pronounced side effects, including photophobia, impaired near vision, and increased intraocular pressure (IOP) (12–14). Atropine at 1% concentration is used in clinical practice for pharmacological dilation of the pupil and paralysis of the ciliary muscle (15, 16). The use of low-concentration topical atropine for myopia control in children is linked to certain ocular AEs, such as mydriasis, photophobia, and reduced accommodation. These effects may manifest as symptoms of glare and blur, specifically during activities involving close visual work (17, 18). Though typically of a mild nature, subjective side effects have the potential to impede academic and outdoor pursuits, thus serving as a notable factor contributing to the discontinuation of treatment.

Polling et al. documented that the most negative occurrences were photophobia (72%), followed by difficulties with reading (38%), and headaches (22%) when 0.5% atropine was used to treat myopic children (19, 20). In contrast, side effects such as photophobia can be resolved by outdoor use of sunglasses in children (15, 21). On the other hand, reports suggest that 0.01% atropine had no significant AEs on contract sensitivity in myopic adults (22). As daily use of low concentrations of atropine is recommended for children undergoing myopia control therapy, there is concern about the potential development of dry eye with prolonged atropine administration (23, 24). Therefore, the current study is intended to assess the short-term changes in ocular surface parameters, pupil size, and quality of vision in young adults treated with 0.01% concentration of atropine.

## 2 Methods

### 2.1 Study design and participants

This investigation was conducted under the supervision of the Institutional Review Committee of He Eye Hospital in Shenyang, China, as per the Declaration of Helsinki (approval number: IRB (2022) K011.01) and registered with ClinicalTrials.gov (NCT06071260). After thoroughly explaining the nature of the study and its potential repercussions, written consent was obtained from every participant. Participants’ information was collected between 1 December 2023 to 20 December 2023. Thirty-three adults (66 eyes) were recruited for the study. All the participants met the following inclusion and exclusion criteria.

Inclusion criteria comprised the following: (i) participants who provided consent; (ii) age ≥ 18 years old; (iii) IOP between 10 and 21 mmHg; (iv) refractive error < −6.00 diopter (D) and astigmatism ≤1.50D; and (v) best-corrected visual acuity (BCVA) of 20/20 or above. Exclusion criteria: (i) any keratopathy, fundus lesion; (ii) strabismus, amblyopia, or eye muscle dysfunction caused by any reason; (iii) previous eye surgery; (iv) refusal to use topical 0.01% atropine eye drop; (v) exposure to any ocular drug within the past 30 days; (vi) allergic to atropine; and (vii) inability to follow the examiner’s request.

### 2.2 Patients and public involvement

Patients or the public were not involved in the design, conduct, reporting, or dissemination plans of our research.

### 2.3 Experimental design

**Figure 1:**
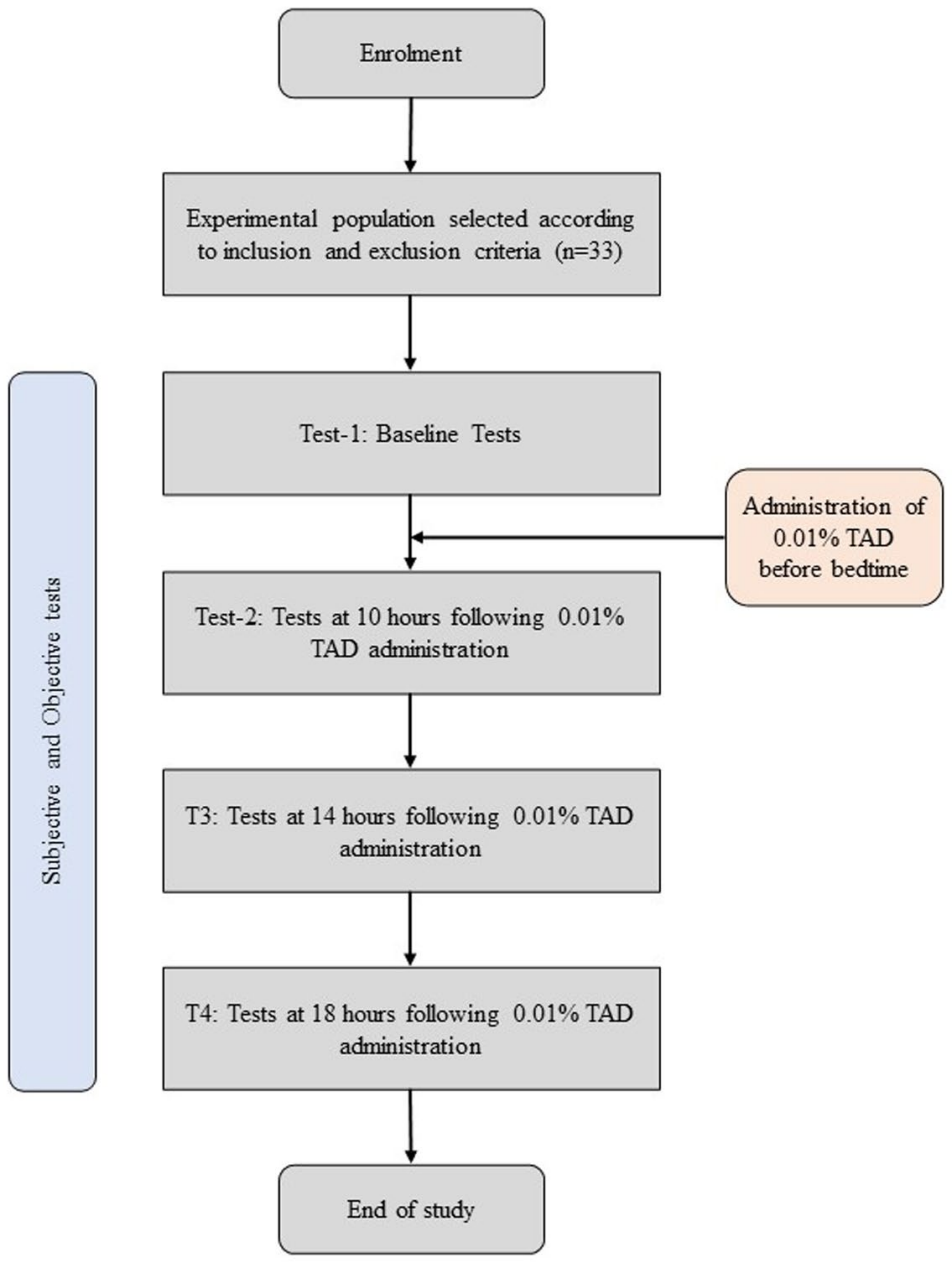
Study flowchart.

This single-arm self-control study assessed participants before and after administering one drop of 0.01% atropine in each eye. Each 3 mL vial of eye drops contains atropine (0.01%), sodium chloride (0.9%), and benzalkonium chloride (0.005%) in an aqueous solution. On day 1, baseline measurements were assessed by a trained clinician; participants were sent back with a bottle of 0.01% topical atropine drop (TAD) and instructed to put one drop in each eye and mark the time on their diary. On day 2 (the following day), 10 h, 14 h, and 18 h following the use of 0.01% TAD, subjective and objective tests were performed by a trained clinician (Figure 1).

### 2.4 Assessments

The OPD Scan III assessed all mesopic pupil diameter (MPD), photopic pupil diameter (PPD), and higher order aberration (HOA) by the same ophthalmologist in the same dark room setting (0.63 lux) (14). All the participants were required to stay in the darkroom for at least 5 min to adapt to the dark condition before the examination. Then, the test was performed 3 times, and the mean value was recorded (25, 26).

The Keratograph 5 M Topographer (OCULUS, Germany) assessed the tear film stability. Parameters of the ocular surface included non-invasive tear breakup time (NITBUT), tear meniscus height (TMH), and RS (redness score) (27, 28).

The quality of vision (QOV) questionnaire consists of (i) symptom frequency, (ii) severity, and (iii) bothersomeness for (a) glare, (b) halo, (c) starbursts, (d) hazy vision, (e) blurred vision, (f) distortion, (g) multiple images, (h) visual fluctuations, (i) difficulty focusing, and (j) difficulty judging distance or depth perception. Respondents use a scale of 0 to 3, where zero (0) denotes “none,” one (1) “a little,” two (2) “quite a bit,” and three (3) “very much.” Furthermore, on a scale from 0 to 10, patients were asked to provide Likert ratings for “daytime” and “nighttime” quality of vision (0 denoting the poorest vision, while 10 indicating the best vision) (29, 30).

The ocular surface disease index (OSDI) questionnaire is widely used to assess the dry eye symptoms of participants. It includes 12 items, divided into three parts (items 1–5 refer to ocular pain and visual difficulties; items 6–9 are about visual functionality; and items 10–12 assess environmental factors). The total OSDI score can range from 0 to 100, with the higher score indicative of worse symptoms of dry eye (31).

### 2.5 Statistical analysis

Statistical Package for Social Sciences (SPSS, version 26, IBM Corp, United States) for macOS software was used to analyze the data. Shapiro–Wilk tests were performed to determine whether the data were normally distributed. A chi-square test was performed on the counting data, and a repeated-measures analysis of variance (ANOVA) was used to evaluate changes before and after medication. An analysis was performed between before treatment and after treatment. A *p-*value of <0.05 was considered statistically significant.

## 3 Results

### 3.1 Patient characteristics

Table 1 displays the demographic characteristics of the study participants. The mean standard deviation (SD) age of the 33 participants (22 women and 11 men) was 24.91 ± 3.36 (range: 21 to 35 years), mean spherical equivalent were − 3.78 ± 2.33 diopters, mean cylinders were − 0.72 ± 0.7 diopters, and the BCVA was 1 ± 0.03 (decimal notation).

**Table 1:** Demographic characteristics of participants in the study.

Variable	
Sex, female (%)	22 (66.7%)
Age, mean ± SD (years)	24.91 ± 3.36
BCVA (Decimal notation)	1.0 ± 0.03
SE	−3.78 ± 2.33
C	−0.72 ± 0.7

SD, standard deviation; BCVA, best-corrected visual acuity; SE, spherical equivalent; C, cylinder.

### 3.2 Objective parameter index

Table 2 displays the PPD at baseline was 4.78 ± 0.75 mm, and following the administration of 0.01% atropine, PPD was found to be 5.83 ± 0.88 mm (*p* < 0.001) at 10 h, 5.52 ± 0.86 mm (*p <* 0.001) at 14 h, and 5.12 ± 0.91 mm (*p <* 0.005) at 18 h and were all significantly larger than baseline measurements. The MPD at baseline was 6.72 ± 0.81 mm. After administration of 0.01% atropine, the PPD was 7.25 ± 0.74 mm (*p* < 0.001) at 10 h, 7.02 ± 0.92 mm (*p <* 0.005) at 14 h, and 6.87 ± 0.80 mm (*p <* 0.244) at 18 h. Measurements at 10 h and 14 h were significantly larger than the baseline measurements (Table 2).

**Table 2:** Characteristics of participants at baseline and after using 0.01% atropine.

Groups		Test 1 (Baseline)	Test 2 (10 h)	*p*-value^1^	Test 3 (14 h)	*p*-value^2^	Test 4 (18 h)	*p*-value^3^
Pupil size	Photopic	4.78 ± 0.75	5.83 ± 0.88	0.0001**	5.52 ± 0.86	0.0001**	5.12 ± 0.91	0.005**
	Mesopic	6.72 ± 0.81	7.25 ± 0.74	0.0001*	7.02 ± 0.92	0.05*	6.87 ± 0.80	0.244
HOA		2.65 ± 2.36	2.42 ± 1.23	0.952	2.44 ± 1.19	0.941	2.38 ± 1.17	0.897
NITBUT-F		9.47 ± 5.9	8.14 ± 6.27	0.586	8.75 ± 7.18	0.976	8.31 ± 5.90	0.621
NITBUT-A		14.14 ± 12.07	10.50 ± 6.31	0.144	10.88 ± 5.48	0.204	10.95 ± 6.09	0.252
TMH		0.20 ± 0.07	0.17 ± 0.05	0.007*	0.17 ± 0.05	0.002*	0.18 ± 0.05	0.198
RS		1.00 ± 0.26	1.05 ± 0.37	0.747	1.18 ± 0.83	0.378	1.06 ± 0.27	0.211

* *p* < 0.05, ** *p* < 0.001. Test 1: baseline tests; Test 2: tests after 10 h; Test 3: tests after 14 h; Test 4: tests after 18 h. HOA, higher order aberration; NITBUT-F, first non-invasive breakup time; NITBUT-A, average non-invasive breakup time; TMH, tear meniscus height; RS, redness score.

HOA at baseline was found to be 2.65 ± 2.36 μm and remained significantly (*p* > 0.05) unchanged at 10 h (2.42 ± 1.23 μm), 14 h (2.44 ± 1.19 μm), and 18 h (2.38 ± 1.17 μm). First NITBUT at baseline was 9.47 ± 5.9 s, 8.14 ± 6.27 s at 10 h, 8.75 ± 7.18 at 14 h, and 8.31 ± 5.90 s at 18 h. While a decreasing trend was found in the first NITBUT measurements following the use of atropine, all changes were not statistically significant compared to the baseline measurements (*p* > 0.05). Average NITBUT at baseline was 14.14 ± 12.07 s, 10.50 ± 6.31 s at 10 h, 10.88 ± 5.48 at 14 h, and 10.95 ± 6.09 s at 18 h. Similar to the first NITBUT, all changes were not statistically significant when compared to the baseline measurements (*p* > 0.05). TMH at baseline, at 10 h, 14 h, and 18 h was recorded to be 0.20 ± 0.07 mm, 0.17 ± 0.05 mm, 0.17 ± 0.05 mm, and 0.18 ± 0.05 mm, respectively. Statistically significant differences were found when comparing the baseline with 10 h (*p* = 0.007) and the baseline with 14 h (*p* = 0.002). Finally, we compared the variations in RS prior to and following the use of eye drops and found no significant difference (*p* > 0.05) (Table 2).

### 3.3 Subjective parameter index

In the QOV questionnaire, there was a statistically significant difference between patients before and 10 h and 14 h after receiving 0.01% atropine (*p* < 0.001, *p* = 0.003). After 18 h, there was no significant difference (*p* = 1.00) (Table 3).

**Table 3:** Characteristics of participants at baseline and after using 0.01% atropine.

Groups	Test 1 (Baseline)	Test 2 (10 h)	*p* ^1^	Test 3 (14 h)	*p* ^2^	Test 4 (18 h)	*p* ^3^
Glare	0.52 ± 1.06	1.55 ± 2.42	0.004*	1.33 ± 2.10	0.003*	0.70 ± 1.23	0.744
Haloes	0.21 ± 0.73	0.36 ± 1.05	0.932	0.15 ± 0.56	0.996	0.12 ± 0.54	0.967
Starbursts	0.39 ± 1.14	0.64 ± 1.57	0.801	0.45 ± 1.19	0.999	0.30 ± 1.01	0.976
Hazy	0.79 ± 1.03	1.94 ± 2.61	0.003*	1.27 ± 1.74	0.085	0.64 ± 1.19	0.888
Blurred	0.97 ± 1.30	2.33 ± 2.57	0.0001**	1.64 ± 1.94	0.035*	1.09 ± 1.41	0.965
Distortion	0.00 ± 0.00	0.12 ± 0.69	0.646	0.00 ± 0.00	–	0.00 ± 0.00	–
Multiple vision	0.39 ± 0.89	0.73 ± 1.41	0.244	0.24 ± 0.61	0.799	0.30 ± 0.76	0.980
Fluctuation	0.27 ± 0.76	0.64 ± 1.21	0.023*	0.36 ± 0.812	0.943	0.18 ± 0.52	0.857
Focusing difficulties	0.55 ± 1.03	2.55 ± 2.38	0.0001**	1.58 ± 1.67	0.0001**	0.91 ± 1.58	0.479
Depth perception	0.00 ± 0.00	0.15 ± 0.61	0.261	0.06 ± 0.35	0.646	0.00 ± 0.00	–
Day	9.03 ± 1.00	7.52 ± 2.35	–	8.27 ± 1.43	–	8.55 ± 1.51	–
Night	8.67 ± 1.00	–	–	–	–	–	–

* *p* < 0.05, ** *p* < 0.001. Test 1: baseline tests; Test 2: tests after 10 h; Test 3: tests after 14 h; Test 4: tests after 18 h. P1, *p*-value for Baseline vs. 10 h; P2, *p*-value for baseline vs. 14 h; P3, *p*-value for baseline vs. 18 h.

We then evaluated the prevalence, severity, and impact of specific visual phenomena prior to and following atropine administration. The results for the symptoms reported by patients 10 h and 14 h after atropine use differed significantly from those reported before atropine use: glare (*p* = 0.004 10 h and *p* = 0.003 14 h), blurred vision (*p* < 0.0001 10 h and *p* = 0.035 at 14 h), and focusing difficulties (*p* < 0.000110 h and *p* < 0.0001 14 h). Additionally, there were significant differences in hazy vision and vision fluctuation before and 10 h after atropine use (*p* = 0.003 and *p* = 0.023, respectively) (Table 3; Supplementary Figure S1).

In order to assess the change in subjective dry eye symptoms before and after the use of 0.01% atropine eye drops, the OSDI questionnaire was administered at baseline, after 10 h, 14 h, and 18 h. According to the findings (Table 4), there was a statistically significant difference between 0.01% atropine 10 h after usage and before use (*p* < 0.001).

**Table 4:** OSDI questionnaire scores at different time.

Groups	Overall score
Test 1 (Baseline)	11.21 ± 11.52
Test 2 (10 h)	27.41 ± 21.11
*p-*value^1^	0.0001**
Test 3 (14 h)	19.58 ± 16.97
*p*-value^2^	0.001**
Test 4 (18 h)	13.04 ± 11.90
*p*-value^3^	0.667

* *p* < 0.05, ** *p* < 0.001. Test 1: baseline tests; Test 2: tests after 10 h; Test 3: tests after 14 h; Test 4: tests after 18 h.

## 4 Discussion

This study aimed to examine the immediate impact of administering 0.01% atropine drops on both the ocular surface and visual quality. The findings indicate that a single dose (one drop in each eye) of 0.01% atropine had a discernible impact on the ocular surface and quality of vision in adult patients with myopia that lasted overnight and returned to initial values after 18 h. While there is no notable disparity in the impact on the NITUBT and RS scores. The study observed a significant rise in dry eye symptoms, particularly within 8 h of dosing. However, these symptoms returned to their initial levels after 18 h. Moreover, the size of the pupil (in both the presence of light and darkness) showed a significant increase. In particular, even after 18 h, the pupil size in the presence of light remained significantly different from the initial measurement. The symptoms of QOV, including difficulty in focusing, glare, and blurred vision, remained for a duration of 14 h and thereafter faded after 18 h. The symptoms of hazy vision and fluctuation in vision persisted for a duration of 8 h.

The use of topical 0.01% atropine eye drops in clinical practice primarily focuses on mitigating the progression of myopia (22, 32–35). Studies have successfully demonstrated the efficacy of atropine impeding the AL growth in myopic children (36, 37). While the endpoint goals of such studies typically focus on monitoring AL growth and visual acuity, they often do not specifically assess the subjective quality of vision and dry eye symptoms. Although some studies have reported complications or AEs such as photophobia, blurred near vision, eye irritation/discomfort, allergic reactions, headache, stye/chalazion, glare, and dizziness, these are generally characterized as complications or AEs (38).

Currently, there is no definitive conclusion on whether long-term use of atropine in children affects the meibomian glands and tear film, thereby causing dry eye. Animal experiments have shown that 1% atropine eye drops can quickly induce dry eye symptoms in rabbit eyes, this effect is weakened after a few weeks (39). However, the abovementioned articles did not include human subjects nor were ocular surface and visual quality indicators assessed.

Patients typically utilize low-concentration atropine for months or even years for myopia control. The current study only observed the changes in the eye within 1 day of the patient’s use. While long-term use is a rational way to validate the effects of low-concentration atropine. Therefore, from the current study, we cannot fully ascertain whether the short-term effects found in this study will persist in real-world settings. According to Luo et al. (40), adult users of 0.01% atropine for 14 days found notable variations between the NITBUT-first and NITBUT-average. On the other hand, the differences receded when the treatment was stopped, which could be related to the low concentration of the atropine eye drop. However, prolonged applications (years) could lead to cumulative or transient effects. Therefore, future studies will aim to assess long-term use and quality of vision changes (41).

The research is limited by the small sample size of the trial and by the fact that data were collected at only one site. The attainment of statistical significance with a limited sample size for both indications and symptoms underscores the importance of the findings (41). Moreover, the present study specifically examined myopic individuals in their youth, rather than children in general. As a result, these findings cannot be extrapolated to the broader population of children. It is evident that younger individuals may exhibit similar indications and symptoms as adults (42, 43). However, adults are likely to provide a more discerning evaluation of the side effects compared to children. Nevertheless, subsequent investigations will focus on expanding the sample size, including younger individuals, and implementing a randomized controlled approach. Furthermore, formulations with low amounts of atropine commonly include BAK as a preservative. It is important to note that Benzalkonium chloride (BAK) is known to be harmful to the corneal epithelium and has been linked to the development of dry eye syndrome. Several investigations have specifically emphasized the harmful effects of it on the retinal tissue (44, 45). While preservative-free low-dose atropine is commercially not available, future studies will aim to compare preservative-free and BAK low-dose atropine drops (17, 46).

## 5 Conclusion

The findings in this research support the notion that a low concentration of atropine (0.01%) temporarily impacts both subjective and objective dry eye tests and subjective visual quality. However, these evaluations revert to their original levels approximately 18 h after their application. This finding may suggest how persistent changes to the ocular surface and quality of vision might impact children and adolescents. Ultimately, more investigation and longer follow-up examinations are necessary for myopic patients who undergo long-term therapy with low-concentration atropine.

## Data Availability

The raw data supporting the conclusions of this article will be made available by the authors, without undue reservation.

## Supplementary material

SUPPLEMENTARY FIGURE S1 Change in QoV questionnaire score at various time points.

## References

[1] Spillmann L. Stopping the rise of myopia in Asia. Graefes Arch Clin Exp Ophthalmol. (2019) 258:943–59. doi: 10.1007/S00417-019-04555-0 31873785

[2] Morgan IG Wu PC Ostrin LA Tideman JW Yam JC Lan W. IMI risk factors for myopia. Invest Ophthalmol Vis Sci. (2021) 62:3. doi: 10.1167/iovs.62.5.3, PMID: 33909035 PMC8083079

[3] Hessler P Künzel P Degle S. Comparison of three different devices for the evaluation of axial length, refractive error, and Keratometry. Optom Vis Sci. (2023) 100:557–63. doi: 10.1097/OPX.0000000000002022, PMID: 37097987 PMC10510797

[4] Celorio JM Pruett RC. Prevalence of lattice degeneration and its relation to axial length in severe myopia. Am J Ophthalmol. (1991) 111:20–3. doi: 10.1016/S0002-9394(14)76891-6, PMID: 1985485

[5] Liu Z Pazo EE Ye H Yu C Xu L He W. Comparing school-aged refraction measurements using the 2WIN-S portable refractor in relation to Cycloplegic retinoscopy: a cross-sectional study. J Ophthalmol. (2021) 2021:6612476. doi: 10.1155/2021/6612476, PMID: 34094595 PMC8163555

[6] Lawrenson JG Shah R Huntjens B Downie LE Virgili G Dhakal R. Interventions for myopia control in children: a living systematic review and network meta-analysis. Cochrane Database Syst Rev. (2023) 2:CD014758. doi: 10.1002/14651858.CD014758.pub2 36809645 PMC9933422

[7] Moriche-Carretero M Revilla-Amores R Gutiérrez-Blanco A Moreno-Morillo FJ Martinez-Perez C Sánchez-Tena MÁ. Five-year results of atropine 0.01% efficacy in the myopia control in a European population. Br J Ophthalmol. (2023) 108:715–9. doi: 10.1136/BJO-2022-322808 37268328

[8] Zhao C Cai C Ding Q Dai H. Efficacy and safety of atropine to control myopia progression: a systematic review and meta-analysis. BMC Ophthalmol. (2020) 20:1–8. doi: 10.1186/S12886-020-01746-W 33287746 PMC7720573

[9] Xu S Li Z Zhao W Zheng B Jiang J Ye G. Effect of atropine, orthokeratology and combined treatments for myopia control: a 2-year stratified randomised clinical trial. Br J Ophthalmol. (2023) 107:1812–7. doi: 10.1136/bjo-2022-321272, PMID: 36229177

[10] Qin G Chen J Hu L Qi Y Xu L He W. Protocol for a parallel assignment prospective, randomised, double-blinded, placebo-controlled trial to evaluate the efficacy of 0.01% atropine for near work-induced transient myopia and myopic progression in China. BMJ Open. (2023) 13:e079833. doi: 10.1136/bmjopen-2023-079833, PMID: 38128934 PMC10748874

[11] North RV Kelly ME. A review of the uses and adverse effects of topical administration of atropine. Ophthalmic Physiol Opt. (1987) 7:109–14. doi: 10.1111/j.1475-1313.1987.tb01004.x, PMID: 2958765

[12] Yam JC Li FF Zhang X Tang SM Yip BHK Kam KW. Two-year clinical trial of the low-concentration atropine for myopia progression (LAMP) study: phase 2 report. Ophthalmology. (2020) 127:910–9. doi: 10.1016/j.ophtha.2019.12.011, PMID: 32019700

[13] Azuara-Blanco A Logan N Strang N Saunders K Allen PM Weir R. Low-dose (0.01%) atropine eye-drops to reduce progression of myopia in children: a multicentre placebo-controlled randomised trial in the UK (CHAMP-UK) – study protocol. Br J Ophthalmol. (2020) 104:950–5. doi: 10.1136/bjophthalmol-2019-314819, PMID: 31653669

[14] Chia A Lu QS Tan D. Five-year clinical trial on atropine for the treatment of myopia 2 myopia control with atropine 0.01% Eyedrops. Ophthalmology. (2016) 123:391–9. doi: 10.1016/j.ophtha.2015.07.004, PMID: 26271839

[15] Tran HDM Tran YH Tran TD Jong M Coroneo M Sankaridurg P. A review of myopia control with atropine. J Ocul Pharmacol Ther. (2018) 34:374–9. doi: 10.1089/jop.2017.0144, PMID: 29715053

[16] Clark AJ. The antagonism of acetyl choline by atropine. J Physiol. (1926) 61:547. doi: 10.1113/jphysiol.1926.sp002315, PMID: 16993814 PMC1514864

[17] Chierigo A Desideri LF Traverso CE Vagge A. The role of atropine in preventing myopia progression: an update. Pharmaceutics. (2022) 14:900. doi: 10.3390/PHARMACEUTICS14050900 35631486 PMC9147984

[18] Kothari M Jain R Khadse N Rathod V Mutha S. Allergic reactions to atropine eye drops for retardation of progressive myopia in children. Indian J Ophthalmol. (2018) 66:1446. doi: 10.4103/IJO.IJO_165_18 30249831 PMC6173048

[19] Polling JR Kok RGW Tideman JWL Meskat B Klaver CCW. Effectiveness study of atropine for progressive myopia in Europeans. Eye. (2016) 30:998–1004. doi: 10.1038/eye.2016.78 27101751 PMC4941076

[20] Wang CH Song LJ Yang Y Qu XP Lan L Liu B. Case report: multiple seizures after a Diphenoxylate-atropine overdose in a small child. Front Pharmacol. (2021) 12:646530. doi: 10.3389/fphar.2021.795205, PMID: 33912057 PMC8072351

[21] Fu A Stapleton F Wei L Wang W Zhao B Watt K. Effect of low-dose atropine on myopia progression, pupil diameter and accommodative amplitude: low-dose atropine and myopia progression. Br J Ophthalmol. (2020) 104:1535–41. doi: 10.1136/bjophthalmol-2019-315440, PMID: 32086237

[22] Cheng Z Mei J Cao S Zhang R Zhou J Wang Y. The effects of 0.01% atropine on adult Myopes’ contrast sensitivity. Front Neurosci. (2021) 15:624472. doi: 10.3389/fnins.2021.624472, PMID: 33679306 PMC7933202

[23] Zhao F Ma JX. Will the long-term use of atropine eye drops in children increase the risk of dry eye? Med Hypotheses. (2019) 132:109331. doi: 10.1016/j.mehy.2019.109331, PMID: 31421421

[24] Cristaldi M Olivieri M Pezzino S Spampinato G Lupo G Anfuso CD. Atropine differentially modulates ECM production by ocular fibroblasts, and its ocular surface toxicity is blunted by colostrum. Biomedicines. (2020) 8:78. doi: 10.3390/BIOMEDICINES8040078 32260532 PMC7236597

[25] Mcginnigle S Naroo SA Eperjesi F. Evaluation of the auto-refraction function of the Nidek OPD-scan III. Clin Exp Optom. (2014) 97:160–3. doi: 10.1111/cxo.12109, PMID: 24024877

[26] Zhang Q Wu Y Huang H Qin G Li L Chen J. The influence of pupil diameter upon and subjective quality of vision following implantable collamer lens (ICL V4c) implantation: an observational study. Medicine. (2023) 102:E35198. doi: 10.1097/MD.0000000000035198 37800803 PMC10553097

[27] Best N Drury L Wolffsohn JS. Clinical evaluation of the Oculus Keratograph. Cont Lens Anterior Eye. (2012) 35:171–4. doi: 10.1016/j.clae.2012.04.002, PMID: 22542606

[28] Tian L Qu J Zhang X Sun X. Repeatability and reproducibility of noninvasive Keratograph 5M measurements in patients with dry eye disease. J Ophthalmol. (2016) 2016:1–6. doi: 10.1155/2016/8013621, PMID: 27190639 PMC4844888

[29] McAlinden C Pesudovs K Moore JE. The development of an instrument to measure quality of vision: the quality of vision (QoV) questionnaire. Invest Ophthalmol Vis Sci. (2010) 51:5537–45. doi: 10.1167/iovs.10-5341, PMID: 20505205

[30] Fan Q Pazo EE You Y Zhang CC Zhang CC Xu L. Subjective quality of vision in evaporative dry eye patients after intense pulsed light. Photobiomodul Photomed Laser Surg. (2020) 38:444–5. doi: 10.1089/photob.2019.4788 32357083

[31] Zhang X Yang L Zhang Q Fan Q Zhang C You Y. Reliability of Chinese web-based ocular surface disease index (C-OSDI) questionnaire in dry eye patients: A randomized, crossover study. Int J Ophthalmol. (2021) 14:834–43. doi: 10.18240/ijo.2021.06.07 34150537 PMC8165610

[32] Yu M Jiang L Chen M. Effect of atropine 0.01% on myopia control in children aged 6–13 years during the 2022 lockdown in Shanghai. Front Public Health. (2023) 11:1074272. doi: 10.3389/fpubh.2023.1074272, PMID: 36778567 PMC9909278

[33] Gan J Li SM Wu S Cao K Ma D He X. Varying dose of atropine in slowing myopia progression in children over different follow-up periods by Meta-analysis. Front Med (Lausanne). (2022) 8:756398. doi: 10.3389/FMED.2021.756398/BIBTEX 35096861 PMC8792607

[34] Wang M Cui C Yu SA Liang LL Ma JX Fu AC. Effect of 0.02 and 0.01% atropine on ocular biometrics: a two-year clinical trial. Front Pediatr. (2023) 11:1095495. doi: 10.3389/FPED.2023.1095495/BIBTEX 36733432 PMC9888550

[35] Hou P Wu D Nie Y Wei H Liu L Yang G. Comparison of the efficacy and safety of different doses of atropine for myopic control in children: a meta-analysis. Front Pharmacol. (2023) 14:1227787. doi: 10.3389/FPHAR.2023.1227787/BIBTEX 37767401 PMC10520549

[36] Gong Q Janowski M Luo M Wei H Chen B Yang G. Efficacy and adverse effects of atropine in childhood myopia a meta-analysis. JAMA Ophthalmol. (2017) 135:624–30. doi: 10.1001/jamaophthalmol.2017.1091, PMID: 28494063 PMC5710262

[37] Yam JC Jiang Y Tang SM Law AKP Chan JJ Wong E. Low-concentration atropine for myopia progression (LAMP) study: a randomized, double-blinded, placebo-controlled trial of 0.05, 0.025, and 0.01% atropine eye drops in myopia control. Ophthalmology. (2019) 126:113–24. doi: 10.1016/j.ophtha.2018.05.029, PMID: 30514630

[38] Sun H Bu F Xin X Yan J. Incidence of adverse events induced by atropine in myopic children: a meta-analysis. J Clin Pharmacol. (2023) 63:1377–86. doi: 10.1002/jcph.2320, PMID: 37492894

[39] Sánchez-Ríos A Correa-Gallegos EY Medina-Espinoza JM Navarro-Sanchez AA Olvera-Montaño O Baiza-Durán L. Validation of a preclinical dry eye model in New Zealand white rabbits during and following topical instillation of 1% ophthalmic atropine sulfate. Animal Model Exp Med. (2022) 5:266–73. doi: 10.1002/ame2.12218, PMID: 35277950 PMC9240734

[40] Luo Y Yin Z Zhang J Wang W Huang Y Li X. Differential impact of 0.01 and 0.05% atropine Eyedrops on ocular surface in Young adults. Transl Vis. Sci Technol. (2024) 13:22–2. doi: 10.1167/tvst.13.4.22, PMID: 38625083 PMC11033597

[41] Wang MTM Muntz A Mamidi B Wolffsohn JS Craig JP. Modifiable lifestyle risk factors for dry eye disease. Cont Lens Anterior Eye. (2021) 44:101409. doi: 10.1016/j.clae.2021.01.004, PMID: 33485806

[42] Upadhyay A Beuerman RW. Biological mechanisms of atropine control of myopia. Eye Contact Lens. (2020) 46:129–35. doi: 10.1097/ICL.0000000000000677, PMID: 31899695 PMC7176345

[43] Cyphers B Huang J Walline JJ. Symptoms and ocular findings associated with administration of 0.01% atropine in young adults. Clin Exp Optom. (2023) 106:311–21. doi: 10.1080/08164622.2022.2033603, PMID: 35188076 PMC9903161

[44] Datta S Baudouin C Brignole-Baudouin F Denoyer A Cortopassi GA. The eye drop preservative benzalkonium chloride potently induces mitochondrial dysfunction and preferentially affects LHON mutant cells. Invest Ophthalmol Vis Sci. (2017) 58:2406–12. doi: 10.1167/iovs.16-20903, PMID: 28444329 PMC5407244

[45] Khanal S Phillips JR. Which low-dose atropine for myopia control? Clin Exp Optom. (2020) 103:230–2. doi: 10.1111/cxo.12967, PMID: 31489714 PMC7065125

[46] Effect of Benzalkonium chloride in corneal permeation of low dose atropine-MedSci.cn. Available at: https://www.medsci.cn/sci/show_paper.asp?id=3caee12693e578b5 (accessed August 17, 2024).

